# DIAGNOdent Pen versus tactile sense for detection of subgingival calculus: an in vitro study

**DOI:** 10.1002/cre2.5

**Published:** 2015-10-05

**Authors:** Fardad Shakibaie, Laurence J Walsh

**Affiliations:** ^1^ School of Dentistry The University of Queensland Brisbane Australia

**Keywords:** Detection, DIAGNOdent Pen, laser fluorescence, subgingival calculus

## Abstract

The objective of this study is to compare the performance of the DIAGNOdent laser fluorescence (LF) Pen to conventional periodontal probing for detection of subgingival calculus under defined laboratory conditions. Extracted teeth with various levels of subgingival deposits of calculus were mounted anatomically in stone casts, and an impression material was used to replicate periodontal soft tissues. The casts were examined for the presence of subgingival calculus at eight surfaces per tooth (240 sites) using LF and a periodontal probe. Sites were rescored after 1 and 3 weeks. Direct imaging of the root surfaces under magnification was the gold standard. As a result, for an experienced operator, LF was more accurate than tactile assessment (across all sites, 84.0% vs. 59.8%). The performance difference was greater for multi‐rooted teeth (85.8% vs. 56.9%) than single‐rooted teeth (77.2% vs. 66.7%). The performance of LF in this laboratory trial was influenced strongly by clinician skill and experience. When used by an experienced operator, LF was more sensitive (75.1% vs. 69.2%), specific (92.6% vs. 86.3%), and accurate (84% vs. 77.9%) than for an inexperienced operator. In conclusion, under the defined laboratory conditions used, LF had better performance than tactile examination, particularly for multi‐rooted teeth. This method may have value clinically as an adjunct for detecting subgingival deposits of calculus in clinical practice. The usefulness of the method improves with operator experience.

Periodontal therapy relies on effective debridement to remove biofilm and dental calculus from teeth and implants. Subgingival deposits of calculus form from calcium phosphate minerals that aggregate within subgingival plaque biofilms (Mandel & Gaffar 1986; White 1997). The rough and porous surfaces of calculus provide a reservoir for periodontopathogenic bacteria (Bird et al. 2001; Shakibaie et al. 2001) and their products (Shirato et al. 1981). Even though effective removal of subgingival deposits of calculus is a cornerstone of periodontal therapy (Cobb 1996; Van der Weijden & Timmerman 2002), this is difficult to achieve in many situations because the deposits are hidden from view (Ånerud et al. 1991; Pippin & Feil 1992).

The conventional method for detecting subgingival calculus is tactile examination using a periodontal probe (Sherman et al. 1990; Pippin & Feil 1992). The tip of the probe should be walked across the root surface, checking for areas of roughness or irregularities (Jones et al. 1972). Tactile examination is prone to false negatives (from burnished calculus that appears smooth to the touch) and to false positives (from instrument‐induced irregularities on the root surface) (Otero‐Cagide & Long 1997; Folwaczny et al. 2004). A false positive result will led to overtreatment, with consequential removal of healthy cementum and dentine, and risks of dentinal hypersensitivity (Haugen & Johansen 1988; Grant et al. 1993; Tammaro et al. 2000). In contrast, with a false negative result, deposits of subgingival calculus will be overlooked, leading to refractory or persistent inflammation. Tactile examination results have limited reproducibility between various operators because they are influenced strongly by clinician skill and experience (Pippin & Feil 1992).

To assist in clinical detection of subgingival calculus, a number of optical methods have been contemplated, including light‐induced fluorescence using lasers (LF). The DIAGNOdent Pen (Kavo, Biberach, Germany) uses 655 nm visible red light to generate near infrared fluorescence emissions from bacterial products, producing an intensity score on a 0–99 scale. While originally promoted as an adjunct to caries detection, the system has applicability to calculus detection because of highly fluorescent porphyrin derivatives in subgingival bacteria and dental calculus, thereby giving a higher score than clean roots (Hibst & Gall 1998; Hibst & Paulus 1999; Hibst & Paulus 2000). Both saliva and blood can influence LF measurements of calculus using the DIAGNOdent (Folwaczny et al. 2002).

The present study was undertaken to compare the effectiveness of LF and tactile examination for detecting subgingival deposits of calculus under defined conditions. Past laboratory studies, which have compared the performance of LF with tactile examination (Folwaczny et al. 2002; Krause et al. 2003), used the older DIAGNOdent Classic system and did not replicate the clinical situation in that the root surfaces were not covered at the time of the assessment. The present study uses a clinical simulation approach with two blinded operators of differing levels of experience with LF systems examining extracted posterior teeth covered with a soft tissue replicant material, with the teeth in a phantom head to simulate the clinical setting (Shakibaie & Walsh 2012; Shakibaie & Walsh 2014).

## Material and Methods

### Model preparation

A total of 30 extracted human posterior teeth (18 molars, 12 premolars) were obtained with the approval of the institutional ethics committee (Reference no: 2003000040), from patients undergoing forceps extraction in a dental school exodontia clinic. The teeth were cleaned with toothbrush under tap water to remove dental plaque and stored in water with 0.1% thymol until mounted. All root surface caries and restorations were removed. The apical third of each tooth root was mounted into one of three stone casts (made from non‐fluorescent dental stone) formed using a mold so that they could later be inserted into a Frasaco phantom head (Frasaco GmbH, Tettnang, Germany). Each model had 10 teeth (four premolars and six molars). The coronal 10–15 mm of each root was left uncovered. To eliminate the possibility of boundary effects from reflection or absorption of light, the interface of the stone and the most apical exposed 2‐mm region of each root was debrided using an ultrasonic scaler to remove any traces of subgingival deposits of calculus. This area was excluded from subsequent analysis.

After application of a non‐fluorescing separator containing water and carboxymethyl cellulose (Oralube artificial saliva), the middle and coronal thirds of the roots were covered with a non‐fluorescing medium bodied silicone impression material (Monet Clearbite 2, Erskine Dental, Sydney) to simulate pocket depths greater than 10 mm. Once set, this material was trimmed with a No. 15 surgical blade to approximate the anatomical contours of gingival tissue. The prepared casts were then soaked in water to ensure maximum hydration of the teeth. The silicone impression material remained in place throughout the LF and tactile examinations.

### Assessment of root surfaces

The models with the mounted teeth were fixed into the phantom head, the flexible face mask applied, and the head positioned in the normal supine operating position at the level of the clinician's elbow. With the aid of a conventional halogen dental operating light, two operators scored each tooth at eight surfaces per tooth (the four line angles and the four intervening middle regions) using a DIAGNOdent Pen (model 2190) fitted with a sapphire periodontal probe (Cat. no. 1.004.1640, KaVo, Biberach, Germany). The DIAGNOdent Pen was calibrated daily to the manufacturer's specifications using a ceramic standard. In all cases, the peak value was used as the final data point for the surface.

One operator was a graduate student with 2 years general dental practice experience who was familiar with the use of LF systems, while the second operator was a final year dentistry student who had not used the DIAGNOdent or other LF systems previously. After scoring all teeth using LF, the models were placed once more into the phantom head (but in a random sequence) and scored for calculus using a William's periodontal probe (Pro 14W) (Hu‐Friedy, Chicago, IL, USA). The LF and tactile readings were recorded separately so that there was no knowledge of previous examination results.

The scoring process was repeated 1 and 3 weeks after the initial assessment in order to evaluate intra‐examiner reproducibility.

### Gold standard

After scoring was complete, the impression material was removed from the models and the root surfaces examined using magnification (from 3 to 20×) using an Olympus U‐PMTVC stereo microscope (Olympus, Tokyo, Japan) fitted with a beam splitter and a 3.3 megapixel digital camera (model Coolpix 995, Nikon, Tokyo, Japan) with composite video output to a high resolution 18‐inch professional monitor. The presence or absence of calculus was scored on eight surfaces per tooth (the four line angles and the four intervening middle regions).

### Data analysis

Sensitivity, specificity, and accuracy were calculated by comparing tactile and LF scores with the gold standard. Probing results, which were recorded as either calculus present or calculus absent, were assigned as being true or false positives, or true or false negatives as appropriate. To dichotomize the LF scores, a threshold value was calculated using the receiver operating characteristic (ROC) curve averaged for both operators. Cut‐off fluorescence scores (1–9, 11 or 20) were selected for plotting the accuracy and ROC curves. A threshold level of 5 was used for generating dichotomized DIAGNOdent Pen results. Separate data sets were generated for comparing the performance of the two methods for single versus multi‐rooted teeth. Changes in the performance of the different methods were combined for weeks 0, 1, and 3, and the data sets for LF and probing were compared using two‐tailed paired *t*‐tests, as data sets met criteria for parametric statistical tests (normal distribution and comparable variances). For the two different operators, the inter‐examiner reliability was calculated using the Cohen kappa statistic.

## Results

### DIAGNOdent Pen threshold level

**Figure 1:**
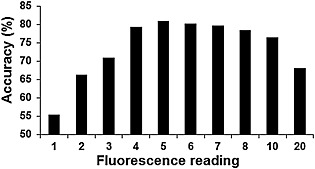
Percentage accuracy for DIAGNOdent Pen at 10 selected laser fluorescence thresholds averaged for both operators. The threshold of 5 gave the best accuracy (80.85%).

**Figure 2:**
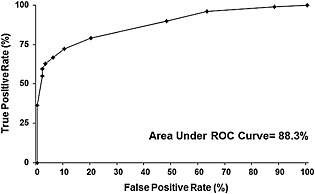
DIAGNOdent Pen receiving operator characteristic (ROC) curve using combined data for both operators.

As shown from ROC analysis, the best performance for LF was obtained by using a threshold fluorescence score of 5 (Fig. 1), which gave the greatest accuracy (80.85%) of the 10 candidate thresholds used. The area under the ROC curve was calculated to be 88.3% (Fig. 2).

### Detection performance

Table 1 presents summary data for the performance of the experienced operator, for whom LF was more sensitive (75.1% vs. 51.7%; *P* = 0.0197), specific (92.6% vs. 67.8%; *P* = 0.0117), and accurate than tactile assessment (84.0% vs. 59.8%; *P* = 0.0143). As shown in Table 2, in the hands of the inexperienced operator, LF was more sensitive (69.2% vs. 50.9%; *P* = 0.0768), specific (86.3% vs. 90.4%; *P* = 0.0528), and accurate than tactile assessment (77.9% vs. 70.7%; *P* = 0.1366). Comparing the two operators, the experienced operator had greater sensitivity (75.1% vs. 69.2%; *P* = 0.0454), but the improvement in specificity (92.6% vs. 86.3%; *P* = 0.2706) and accuracy (84.0% vs. 77.9%; *P* = 0.1134) failed to reach statistical significance.

**Table 1:** Performance of two detection methods for an experienced operator.

Experienced operator	DIAGNOdent Pen (*n* = 5) (%)	Periodontal probe (%)
Sensitivity (baseline)	76.3	50.8
Sensitivity (week 1)	73.7	56.8
Sensitivity (week 3)	75.4	47.5
Mean sensitivity	75.1	51.7
Specificity (baseline)	91.8	63.1
Specificity (week 1)	92.6	73.0
Specificity (week 3)	93.4	67.2
Mean specificity	92.6	67.8
Accuracy (baseline)	84.2	57
Accuracy (week 1)	83.3	64.9
Accuracy (week 3)	84.6	57.4
Mean accuracy	84	59.8

**Table 2:** Performance of two detection methods for an inexperienced operator.

Inexperienced operator	DIAGNOdent Pen (*n* = 5) (%)	Periodontal probe (%)
Sensitivity (baseline)	67.8	40.7
Sensitivity (week 1)	68.6	49.2
Sensitivity (week 3)	71.2	62.7
Mean sensitivity	69.2	50.9
Specificity (baseline)	83.6	87.7
Specificity (week 1)	94.3	96.7
Specificity (week 3)	81.1	86.9
Mean specificity	86.3	90.4
Accuracy (baseline)	75.8	64.2
Accuracy (week 1)	81.7	73
Accuracy (week 3)	76.3	74.8
Mean accuracy	77.9	70.7

Summary data for the effect of root configuration are shown in Table 3. For the experienced operator, the LF method was more accurate than periodontal probing for multi‐rooted teeth (77.2% vs. 85.8%; *P* = 0.0438), whereas the benefit of LF for single‐rooted teeth was too small to reach statistical significance (66.7% vs. 56.9%; *P* = 0.4519). For the inexperienced operator, the accuracy of the LF method was similar to probing for single‐rooted teeth (78.7% vs. 76.6%; *P* = 0.4791).

**Table 3:** Effect of tooth root configuration.

			Single‐rooted (%)	Multi‐rooted (%)
Experienced operator	DIAGNOdent Pen	Mean sensitivity	61.5	79
		Mean specificity	92.8	92.5
		Mean accuracy	77.2	85.8
	PRO 14W	Mean sensitivity	60.2	49.3
		Mean specificity	73.2	64.5
		Mean accuracy	66.7	56.9
Inexperienced operator	DIAGNOdent Pen	Mean sensitivity	65.4	70.3
		Mean specificity	92	82.9
		Mean accuracy	78.7	76.6
	PRO 14W	Mean sensitivity	46.2	52.2
		Mean specificity	94.2	88.2
		Mean accuracy	70.2	70.2

Data for this table are collated across all three assessment rounds.

### Reliability

The summary data presented in Table 4 show that LF was more reliable than tactile probing. LF has moderate reliability, with a mean Cohen kappa (0.49), which lies between 0.41 and 0.60, whereas periodontal probing gave poor strength of agreement, as the mean Cohen kappa (0.19) was less than 0.2.

**Table 4:** Inter‐examiner variation.

Reliability: Cohen kappa	Laser fluorescence (DIAGNOdent Pen, *n* = 5)	Periodontal probe (PRO 14W)
Week 1 K _1,1_	0.4	0.06
Week 2 K _2,2_	0.55	0.26
Week 3 K _3,3_	0.53	0.24
Mean Cohen's kappa	0.49	0.19

## Discussion

The results of the present study, which was conducted under controlled conditions in a laboratory setting, provide some support for the clinical use of LF as an adjunct to conventional probing, because of its greater sensitivity, specificity, accuracy, and reliability, particularly for multi‐rooted teeth where assessment and debridement are more difficult. As could be expected, the benefits are more apparent when the operator has experience and confidence in using the method. Given the small scale of the project and its context, validation of these potential benefits is needed from a clinical trial.

While past studies have described the performance of the DIAGNOdent Pen as an adjunct in the assessment of occlusal caries (Zhu et al. 2012; Teo et al. 2014), the present study is the first assessment of this device for detecting subgingival calculus. Past laboratory studies of calculus detection were conducted with the older DIAGNOdent Classic benchtop system rather than with the handheld Pen system, and used a geometry, which does not approximate the clinical situation. Some past studies have applied a conical tip directly onto the root surfaces of extracted teeth at right angles, a situation which could not be achieved in real world practice (Folwaczny et al. 2002; Krause et al. 2003; Folwaczny et al. 2004). In the current study, the angulation of the tips to the root surfaces was not 90 degrees but rather in the range of 5–15 degrees, because of the constraints applied by the artificial periodontal pockets. This is a better approximation to the clinical setting. The present study could now however replicate fully the physical features of the periodontal pocket and the marginal gingiva, which would be more flexible than the impression material used. Likewise, no blood was present; although, this is not likely to have influenced the pattern of the results based on the outcomes of previous trials of DIAGNOdent calculus detection on the bench in the presence or absence of blood on the root surface being assessed (Krause et al. 2003).

The performance of LF in this laboratory trial was influenced strongly by clinician skill and experience. The present study used a heavy bodied impression material to provide a realistic level of complexity for the operators when accessing sites within deep pockets. Use of an optical method can be very challenging because unlike a stainless steel periodontal probe, while an operator can gain tactile information from the tip, an operator will be reticent to use a substantial force for fear of damaging or fracturing the sapphire tip. This factor may have contributed to the challenges faced by both operators when assessing the depths of the artificial periodontal pockets to assess the root surfaces. The inexperienced operator would have suffered further challenges from their lesser familiarity with periodontal probing around multi‐rooted teeth and their associated furcation regions. The positive results obtained provide some confidence with regards to the clinical usefulness of the method, which could be expected, especially for multi‐rooted teeth, once operators have gained some confidence and experience with the method.

The cut‐off level for the DIAGNOdent Classic is reported to be a fluorescence score of 6.2 (Krause et al. 2003), while the ROC analysis in the present showed the best cut‐off level for the DIAGNOdent Pen was a score of 5. Knowing this threshold is important for clinical decision‐making, for example, the need for additional debridement. In this context, it is important to recognize that the fluorescence scores reflect the surface area and volume of calculus deposits (Shakibaie & Walsh, 2014).

A final point of importance is that as an optical method, LF may be influenced by scatter (e.g. from blood or saliva), and that fluorescence signals can be quenched by oxidants such as hydrogen peroxide or ozonated water used as irrigant fluids with ultrasonic scalers. In the present study, such confounding factors were absent, and only a thin layer of water‐based non‐fluorescing saliva substitute was present. It remains to be seen how much bleeding will affect the reliability of LF when used during periodontal debridement.

### Clinical relevance

#### Scientific rationale for the study

Detection of small deposits of subgingival calculus is challenging in clinical practice. Various methods have been suggested for improving the ability of clinicians to assess subgingival regions of root surface, to aid in effective debridement during periodontal care.

#### Principal findings

In the hands of an experienced operator, the DIAGNOdent Pen was more accurate and reproducible than periodontal probing for detecting calculus deposits under simulated clinical conditions with teeth mounted in an anatomical configuration in a phantom head, with a semi‐rigid impression material replacing the periodontal soft tissues.

#### Practical implications

Based on this trial, under laboratory conditions, using LF may provide benefits for clinicians during periodontal assessment and debridement.

## Conflict of Interest

The authors declare that they have no conflict of interests.

## Funding Information

This study was supported by the Australian Periodontology Research Foundation (APRF) and Australian Dental Research Foundation (ADRF). The study was not funded by the manufacturer of the laser systems used in the study.
